# Longitudinal white matter and cognitive development in pediatric carriers of the apolipoprotein ε4 allele

**DOI:** 10.1016/j.neuroimage.2020.117243

**Published:** 2020-11-15

**Authors:** Justin Remer, Douglas C. Dean, Kewei Chen, Rebecca A. Reiman, Matthew J. Huentelman, Eric M. Reiman, Sean C.L. Deoni

**Affiliations:** a Advanced Baby Imaging Lab, Rhode Island Hospital, Providence, RI, USA; b Department of Pediatrics, Warren Alpert Medical School at Brown University, Providence RI, USA; c Department of Pediatrics, University of Wisconsin–Madison, Madison WI 53705 USA; d Department of Medical Physics, University of Wisconsin–Madison, Madison WI 5305 USA; e Waisman Center, University of Wisconsin–Madison, Madison WI 53705 USA; f Arizona Alzheimer's Consortium, University of Arizona School of Medicine, Tucson and Phoenix AZ, USA; g Banner Alzheimer's Institute, Phoenix, AZ, USA; h University of Arizona College of Medicine, Phoenix, AZ, USA; i Arizona State University, Phoenix, AZ, USA; j Neurogenomics Division, Translational Genomics Research Institute (TGen), Phoenix, AZ, USA; k Maternal, Newborn, and Child Health Discovery and Tools, Bill and Melinda Gates Foundation; Seattle WA, USA

**Keywords:** Pediatric brain development, APOE, Myelin, White matter, Neurodevelopment, Alzheimer's Disease, Cognitive Development, Genetic predisposition to disease, Genetic risk, mcDESPOT, MRI, Radiology

## Abstract

We have previously demonstrated cross-sectional differences in magnetic resonance imaging (MRI) measurements of white matter myelin and gray matter in infants with or without the apolipoprotein ε4 allele, a major genetic risk factor for late-onset Alzheimer's disease (AD). In this study, we sought to compare longitudinal MRI white matter myelin and cognitive-behavioral changes in infants and young children with and without this allele. Serial MRI and cognitive tests were obtained on 223 infants and young children, including 74 ε4 carriers and 149 non-carriers, 2–68 months of age, matched for age, gestational duration, birth weight, sex ratio, maternal age, education, and socioeconomic status. Automated brain mapping algorithms and non-linear mixed models were used to characterize and compare trajectories of white matter myelin and cognitive-behavioral test scores. The APOE ε4 carriers had statistically significant differences in white matter myelin development, in the uncinate fasciculus, temporal lobe, internal capsule and occipital lobe. Additionally, ε4 carriers had a slightly greater rate of development in early learning composite a surrogate measure of IQ representative of expressive language, receptive language, fine motor, and visual skills, but displayed slightly lower non verbal development quotient scores a composite measure of fine motor and visual skills across the entire age range. This study supports the possibility that ε4 carriers have slightly altered rates of white matter and cognitive development in childhood. It continues to raise questions about the role of APOE in human brain development and the relevance of these developmental differences to the predisposition to AD.

## 1 Background

A growing focus in Alzheimer's Disease (AD) research is understanding and characterizing the earliest preclinical structural and functional changes associated with the disorder. In addition to characterizing the earliest progressive changes associated with the preclinical stages of AD, including but not limited to biological measurements of amyloid-β pathology, tau pathology and neurodegeneration, there is an emerging interest in the identification of even earlier, neurodevelopmental changes, some of which may provide a foothold for AD pathology at older ages. As others and we have shown, there is the chance to investigate these neurodevelopmental changes in cognitively unimpaired persons at differential genetic risk for AD (Dean et al., 2014c, Knickmeyer et al., 2014, Reiman et al., 2020, Quiroz et al., 2013).

The apolipoprotein E (APOE) ε4 allele is the major genetic risk factor for developing AD at older ages. This susceptibility gene is found in about one fourth of the general population and about 60% of individuals with AD dementia (Corder et al., 1993, Mahley and Rall, 2000, Strittmatter and Roses, 1995). In cross-sectional studies of infant APOE ε4 carriers and non-carriers, we and others have reported that ε4 carriers are distinguished from non-carriers by differences in magnetic resonance imaging (MRI) measurements of gray matter volumes and white matter myelin content (Dean et al., 2014c, Knickmeyer et al., 2014). Nevertheless, prior cross sectional studies are limited by their inability to examine differential rates of development and fail to examine anatomical differences in pre defined neuroanatomical regions in populations of ε4 carriers. Additionally, no data has been reported on longitudinal differences in cognitive performance between ε4 carriers and non-carriers prior to 5 years of age.

To address these gaps in knowledge, we performed the first longitudinal analysis of brain development in a large cohort of healthy neurotypical infants and young children (2–67 months of age) stratified by presence or absence of at least one APOE ε4 allele. Using mixed effects modeling, we examined and contrasted white matter (WM) maturation profiles, and cognitive ability trends, in ε4 carriers and non-carriers. An MRI pulse sequence, multi-component Driven Equilibrium Single Pulse Observation of T_1_ and T_2_ (mcDESPOT), was used to assess myelination through calculation of the myelin water fraction (MWF), a surrogate measure of myelin content (Deoni et al., 2012, Dean et al., 2014a), and other procedures were used to dampen the sound and permit studies during natural sleep. Cognitive ability was assessed using the Mullen Scales of Early Learning (Mullen, 1995), a standardized tool that provides age-normalized scores of fine and gross motor function, visual function, and expressive and receptive language ability. Our findings suggest that infant and young children with the APOE ε4 allele have small but significant differences in their rates of white matter myelin and cognitive development.

## 2 Methods

### 2.1 Study subjects

**Fig. 1:**
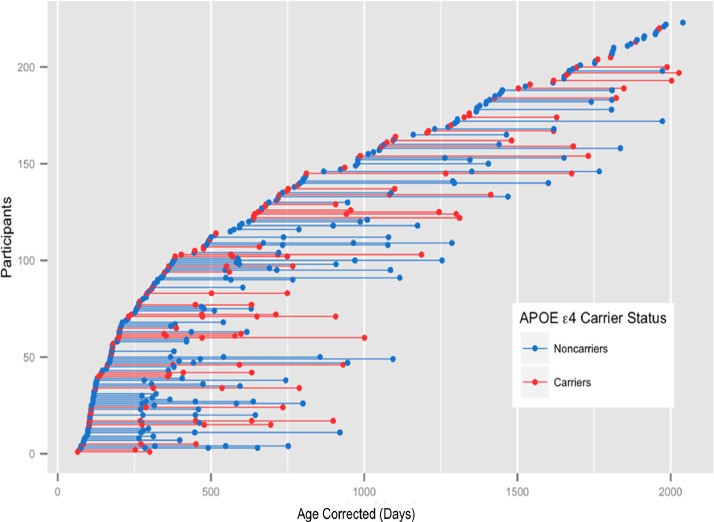
Age distribution of imaging data (red for ε4 carriers are indicated in red, and blue for non-carriers). For a given participant, the age at the initial scan corresponds to the leftmost point on the plot with repeated scans connected by a horizontal line.

Longitudinal MR imaging and cognitive data was obtained from 229 healthy and neurotypical children, 2 months to 5.7 years of age, including 74 APOE ε4 carriers and 149 non-carriers. Six participants were excluded because they had the APOE ε2/ ε4 genotype. In general, children under 2 years of age had MRIs and cognitive assessments at 6-month intervals; while the older children had these procedures yearly. Of the 223 included children, all had at least one MRI; 115 had at least two MRIs; 41 had at least 3 MRIs; and 16 had at least four MRIs. Mean time between repeat scans was 247 days for participants under 2 years of age and 401 days for participants over 2 years of age. (Data from subjects with only a baseline visit was included to extend our prior cross-sectional findings and support assessment of longitudinal changes using mixed modeling (Lindstrom and Bates, 1990). See Fig. 1 for a depiction of the longitudinal design.

Recruitment took place between April 2010 and November 2014 as part of a broader longitudinal study of typical white matter maturation (Deoni et al., 2012, Dean et al., 2014a). Inclusion criteria included: uncomplicated healthy singleton birth between 37 and 42 weeks’ gestation; no abnormalities on fetal ultrasound; no complications during pregnancy; no illicit drug or alcohol use during pregnancy; no admissions to the neonatal intensive care unit; no reported history of neurological events or disorders (e.g. head trauma, epilepsy, etc.); and no familial record of psychiatric or neurological disorder. Criteria were confirmed at time of enrollment through parental interviews. Medical and family history questionnaires were also completed to obtain additional demographic and medical information about the subjects (Table 1). Written consent was obtained from parents or legal guardians in accordance to ethics approval from the host institution's Institutional Review Board. APOE allelic status was determined through polymerase chain-reaction assays on DNA samples obtained from buccal swabs. As stated above participants with the APOE ε2/ ε4 genotype were removed from this study. The child's APOE genotype was withheld from the parents, in accordance to the written consent and institutional review board policies.

**Table 1:** Demographics of study participants.

	Carriers	Non-Carriers	*p*-Value
**Number Of participants**	74	149	
Participants with 2 scans	42	73	
Participants with 3 scans	14	27	
Participants with 4+ scans	6	10	
**APOE Genotype**			
	13 ε4/ε4	120 ε3/ε3	
	61 ε3/ε4	29 ε2/ε3	
**Racial Background**			
African American	10	8	
Asian	0	4	
Caucasian	45	94	
Native American/Alaskan	11	23	
Mixed Race	0	1	
Unknown/Not reported	8	19	
**Mean Age in days (SD)**	770 (53)	750 (564)	0.73
**Gestation in weeks (SD)**	39.4 (1.3)	39.4 (1.2)	0.99
**Female/Male**	33/41	62/87	0.42
**Mean Maternal SES (SD)**	5.0 (2.1)	5.0 (2.0)	0.50
**Mean Maternal Age (SD)**	34.0 (6.3)	31.8 (6.5)	0.26
**Child Feeding (Breast/Bottle/Both/Not reported)**	24/15/19/15	54/27/51/17	0.73
***In-utero*Smoke Exposure (Not Exposed/Exposed/Not reported)**	54/8/12	121/14/14	0.38
**Birth Type (Vaginal/Cesarean/Not reported)**	47/14/13	91/40/18	0.62

Statistical comparisons were performed between APOE ε4 carriers and non-carriers using chi square tests for ethnicity, history of Alzheimer's Disease, race, child feeding, in utero smoke exposure, birth type, gender, and number of scans. *T*-tests compared average age, average gestational period, average maternal age, average maternal SES.

### 2.2 Image acquisition, alignment, and analysis

MRI data were obtained on a Siemens 3 Tesla Tim Trio scanner using a 12 channel head RF coil array. All data was acquired during natural *non-sedated* sleep (for children under 4) or while watching a favorite movie with instructions not to move (Dean et al., 2014b). Subtle body movements were further reduced by swaddling all children with appropriately sized MedVac vacuum immobilization bags (CFI Medical Solutions, USA) Age-optimized mcDESPOT protocols, consisting of 8 *T*_1-_weighted spoiled gradient echo (SPGR) images, 2 inversion-prepared (IR)-SPGR images, and 16 *T*_1_/*T*_2_-weighted balanced steady-state free precision (bSSFP) images, were acquired as detailed previously (Deoni et al., 2012, Deoni et al., 2008). Following acquisition, each child's MWF maps were calculated by fitting the SPGR, (IR)-SPGR, and bSSFP data to a 3-pool tissue model that estimates the volume fractions and relaxation times for the extra/intracellular water, non-exchanging free water, and myelin-associated water (Deoni et al., 2008, Deoni et al., 2013).

To reduce inconsistencies associated with independently registered longitudinal data, a longitudinal registration pipeline was used that first aligns each child's data to his/her own unique subject specific template, created from an average of the acquired time longitudinal time points, and then transfers this to a common study template using nonlinear realignment (Dean et al., 2014a). Registration was performed with the Advanced Normalization Tools (ANTs) software package (Avants et al., 2008), the generated subject specific template, and a *T*_1_-weighted study-specific pediatric brain template in approximate MNI space (Deoni et al., 2012, Dean et al., 2014a, Avants et al., 2008).

(See Supplementary methods for more detail describing image acquisition and registration)

### 2.3 Neuropsychological testing

To evaluate cognitive development across our investigated age-range, the Mullen Scales of Early Learning was used. Cognitive assessments for each child were performed within 1 week of successful MRI. This standardized assessment tool provides age-normalized T-scores for five broad domains, including fine and gross motor function; expressive and receptive language; and visual reception performance (Mullen, 1995). To eliminate known ceiling effects inherent to the Mullen scales (Mullen, 1995, O'Muircheartaigh et al., 2013), data was restricted to subjects under 1500 days (approx. 4 years) of age. In addition to domain-specific measures, the Mullen Scales provides three composite scores, the early learning composite (ELC, derived from the fine motor, visual reception, and expressive and receptive language scores), non-verbal developmental quotient (NVDQ, derived from the fine motor and visual reception scores), and the verbal developmental quotient (VDQ, derived from the expressive and receptive language scores). These three composite scores, which have an age-normalized mean of 100 and standard deviation of 15, were used to examine general cognitive ability (ELC), non-verbal ability (NVDQ), and verbal ability (VDQ), respectively.

### 2.4 Comparison of neuroanatomical region and tract specific MWF development between ε4 carriers and non-carriers

Nonlinear mixed effects modeling (NLMEM) was used to characterize MWF development trajectories for each individual, and the overall population, stratified by APOE allele status (MATLAB 2015b, Natick, MA). NLMEM allows inclusion of both single time point data and multiple measurements per person at different ages, with different or irregular inter-scan intervals (Lindstrom and Bates, 1990). Anatomical masks representing a widespread set of brain regions and pathways were derived from the MNI template and John Hopkins Diffusion Tensor Imaging (DTI)-based white matter atlas (Mori et al., 2009). Each anatomical mask was brought into alignment with our study-specific template that all our subjects were registered to as described previously, and mean MWF values for each neuroanatomical location were obtained for our subjects for each timepoint.

Modified four-parameter Gompertz growth curves (Dean et al., 2014d), were fit to this data using NLMEM. This modified Gompertz model:

MWF(age)=α*exp(−exp(β−γ*age)+η*age)

has been previously shown to provide the most accurate and robust fit for depicting longitudinal myelin development over the 6 years of life (Dean et al., 2014a, 2014d) and is characterized by the parameters: β (the onset of myelination); γ and η (rates of development during early and later developmental stages, respectively); and α (the age at which development shifts from rapid to slow). Individual participant trajectories of neurodevelopment have previously been shown to accurately follow the overall population trajectory in a cohort of this age (see Dean et al. 2015 Figs. 2 and 3) (Dean et al., 2014a, 2014d). For both APOE genotype groups, myelin models were first fit to their respective imaging data. Then for each brain region myelin development parameters were calculated for each APOE genotype group. Both individual trajectories for each participant and overall population models were plotted for both genotype groups. These myelin development parameters (*α, β, γ*, and *η*) as previously stated describe important biologic events in myelin formation and were compared between APOE ε4 carrier and non-carriers using Welch t-tests. Significance for this analysis was defined as *p* < 0.000819 (*p* < 0.05, corrected for 61 brain regions).

### 2.5 Comparison of cognitive development between ε4 carriers and non-carriers

In addition to MWF trajectories, we also examined differences in cognitive measures between the ε4 carriers and non-carriers. Using linear mixed effects modeling, each of the Mullen Scale composite scores (ELC, NVDQ, and VDQ) was modeled as a function of age for both APOE ε4 carrier and non-carriers, with individual variability included as a random effect for both intercepts and slopes. Both individual trajectories for each participant and overall population models were plotted for both genotype groups. A log likelihood ratio test was performed to determine the significance of age as a fixed effect on cognitive development for each of the three Mullen Scale composite scores. Welch t-tests were performed on the intercept and slope of cognitive development trajectories to examine differences between baseline ELC, NVDQ, and VDQ at 0 days, and rate of cognitive development between APOE genotypes respectively. Post hoc analysis involved further comparison of cognitive development trajectories of age normalized T-scores of fine motor, visual reception, expressive language and receptive language. Significance for all cognitive analysis was defined as p < 0.00357 (p < 0.05, corrected for 2 parameters analyzed per model and 7 cognitive metrics). Linear mixed effects modeling was performed using SPSS software (IBM SPSS, Armonk, NY).

### 2.6 Mediation analysis of differential myelin development on differences in cognitive development between ε4 carriers and non-carriers

In order to explore the role of differential myelin development on differential cognitive development a mediation analysis was performed. The Baron and Kenny steps for mediation were performed in order to verify a true mediation relationship. This analysis classified participant genotype as the main independent variable, myelin development parameters as the mediator and cognitive maturation parameters as the dependent variable. The first step involved identifying the cognitive development model parameters that showed a significant relationship to APOE genotype using the process as stated previously. The second step involved identifying the myelin development parameters that showed a significant relationship to APOE genotype as stated previously. Lastly, cognitive development parameters, that showed significant relationships to APOE genotype and myelin development parameters, were then modeled as a function of both APOE genotype and myelin development parameter in order to observe if myelin development parameters mediated the APOE genotype to cognitive development parameter relationships. Of note Myelin developmental trajectories were remodeled for each individual participant using only imaging time points that occurred when participant age was less than 1500 days to line up with the cognitive analysis.

Furthermore, myelin development parameters for each specific anatomical region for each subject along with cognitive development parameters for each Mullen cognitive composite score for each subject were obtained through mixed effects modeling as described previously. Mediation analysis was performed using the python package StatsModels with bootstrapping. Significant myelin model parameter mediation effects on the APOE genotype to cognitive model parameter relationships were defined as defined as *p* < 0.000819 (*p* < 0.05, corrected for 61 brain regions)

## 3 Results

Of the 223 subjects, 74 were identified as APOE ε4 carriers (13 with the ε4/ ε4 genotype and 61 with the ε3/ε4 genotype), and 149 as non-carriers (120 with the ε3/ε3 subjects and 29 with the ε2/ ε3 genotype). A total of 403 MRI images were obtained, with 260 acquired of non-carriers and 143 of ε4 carriers. Carrier and non-carrier groups did not significantly differ in mean participant age, gestation duration, male/female ratio, breast/formula feeding ratio, *in-utero* smoke exposure, vaginal/c-section birth ratio, maternal SES, maternal age, parental marital status, or familial history of AD (Table 1). The number of scans and the mean interval between repeat scans also did not differ significantly between ε4 carriers and non-carriers.

### 3.1 Comparison of region and tract specific MWF development between ε4 carriers and non-carriers

**Fig. 2:**
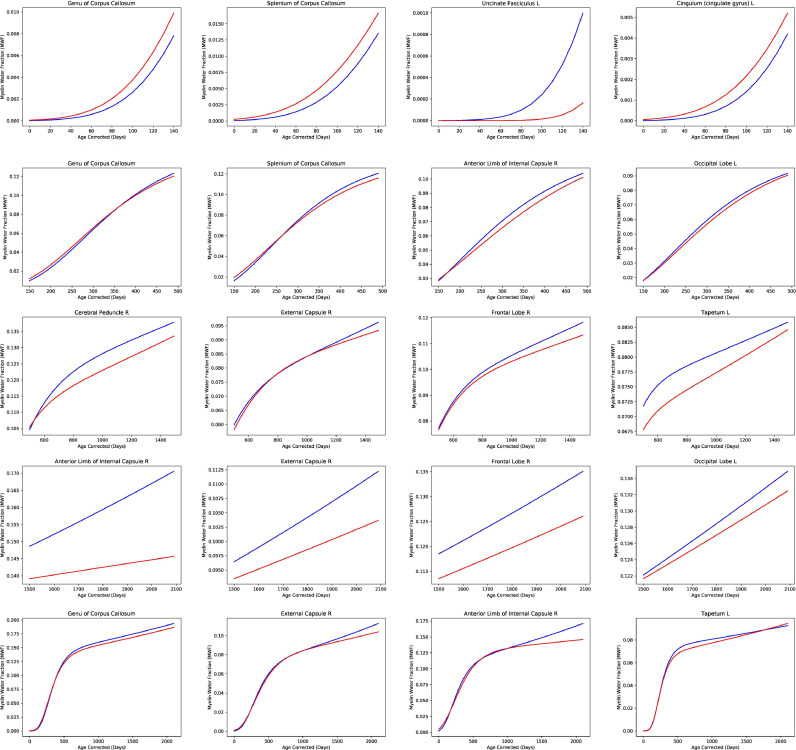
Reconstructed longitudinal MWF trajectories from a representative subset of the examined brain areas. Carrier trajectories are denoted in red, while noncarrier trajectories are displayed in blue. Widespread differences in the trajectories of development were observed between carriers and non-carriers. First row shows differences in the β parameter between ε4 carriers and non-carriers; the second row shows differences in the γ parameter; the third row shows differences in the α parameter; and the fourth row shows differences in η parameter. The figure rows are arranged in chronological order of myelin development events. The last row in the figure shows the overall myelin trajectories for both genotype groups. See Supplementary Table 1 for a complete comparison of the model parameters between ε4 carriers and non-carriers.

**Fig. 3:**
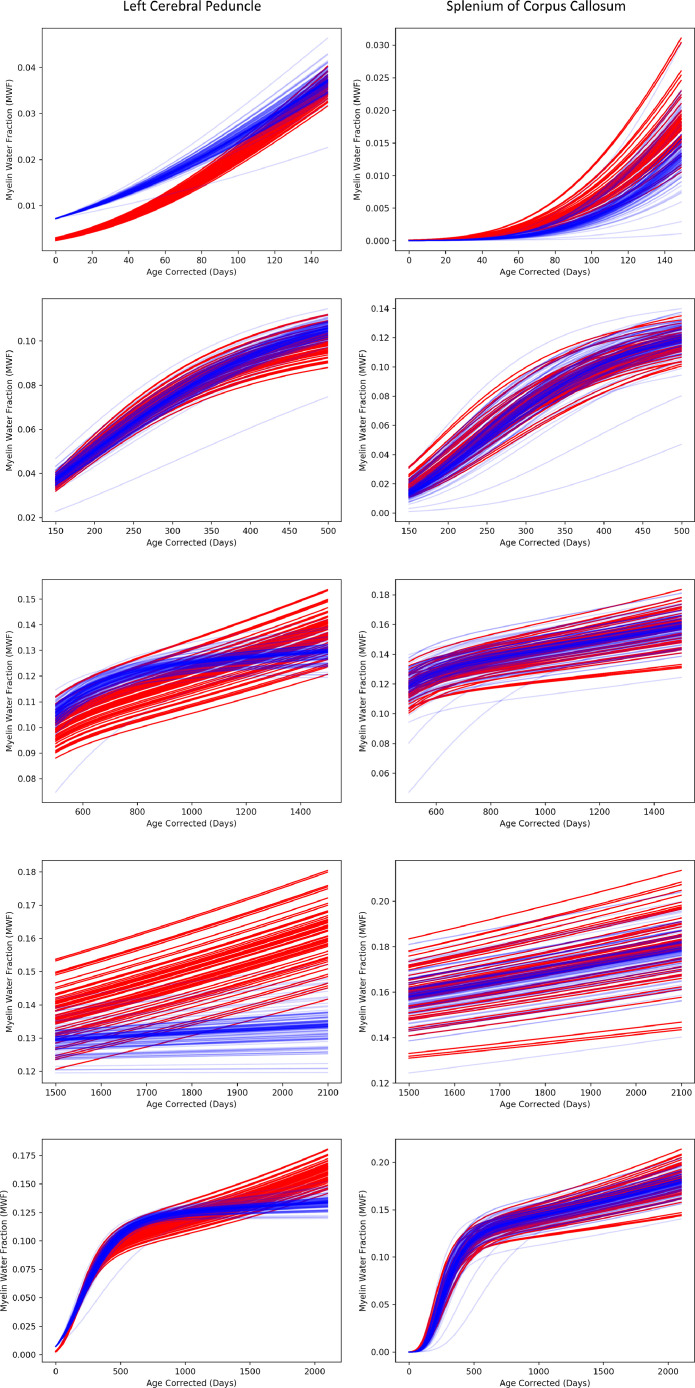
Reconstructed longitudinal MWF trajectories for each participant revealing differential neurodevelopment in the left cerebral peduncle and splenium of the corpus callosum. The red trajectories represent ε4 carriers and the blue trajectories represent non-carriers. The first row shows differences between ε4 carriers and non-carriers in the β parameter, he second row shows differences in the γ parameter; the third row shows differences in the α parameter; and the fourth row shows differences in η parameter. The final row shows the overall MWF trajectories for each participant. Significant differences was observed between all 4 parameters in the left cerebral peduncle; while significant difference in the splenium of the corpus callosum was only observed between the β (first row) γ (second row), and α (third row) parameter.

We found that ε4 carriers and non-carriers showed significantly different patterns of myelination in multiple neuroanatomical locations with respect to all four parameters of early myelin development. Specifically the γ parameter revealed significantly slower rate of early myelination in ε4 carriers multiple brain regions including the genu of corpus callosum (1.83% difference from non-carriers), splenium of corpus callosum (7.15% difference from non-carriers), anterior limb of the internal capsule (25.46% difference from non-carriers), and occipital lobe (9.10% difference from non-carriers). Additionally differences in the η parameter demonstrated significantly slower rates of later myelin development in ε4 carriers in the frontal lobe (9.30% difference from non-carriers), occipital lobe (71.46% difference from non-carriers), external capsule (32.42% difference from non-carriers) and right anterior limb of the internal capsule (67.42% difference from non-carriers). (Figs. 2 and 3, and **Supplementary Table 1**). In contrast, we found ε4 carriers had faster rates of development compared to non-carriers in the right posterior limb of the internal capsule (18.58% difference for γ parameter and 32.23% for η parameter), and right parietal (7.16% difference for γ parameter and 39.63% for η parameter) and right occipital white matter (16.04% difference for γ parameter and 71.46% for η parameter). Examining other aspects of development, specifically the initial onset of myelination (denoted by the β term in the Gompertz model), ε4 carriers showed a significantly earlier onset in the genu and splenium of the corpus callosum (5.42% and 9.70% difference for each region respectively), bilateral anterior limb of the internal capsule (13.94% and 5.30% difference for right and left respectively), bilateral frontal white matter (5.79% and 5.95% difference for right and left respectively), and right uncinate fasciculus (18.26% difference) (Figs. 2 and 3). Non-carriers displayed earlier onset in areas including the bilateral posterior limb of the internal capsule (10.96% difference for both right and left), right cingulum (6.17% difference), left cerebral peduncle (11.88% difference),and left uncinate fasciculus (23.30% difference) (Figs. 2 and 3). Additionally, non carriers display a longer period of early rapid myelin development denoted by significance in α myelin parameter) in multiple brain regions including the left cerebral peduncle (10.65% difference), right external capsule (9.89% difference) and right frontal lobe (2.42% difference). **Supplementary Table 1** provides a complete list of all statistical comparisons between ε4 carriers and non-carriers for all examined brain regions for all 4 myelin development parameters.

### 3.2 Comparison of cognitive measures between ε4 carriers and non-carriers

After removal of data from subjects over 1500 days, and from incomplete assessments, 326 cognitive assessments were obtained consisting of 115 from 59 ε4 carriers; and 211 from 120 non-carriers.

**Fig. 4:**
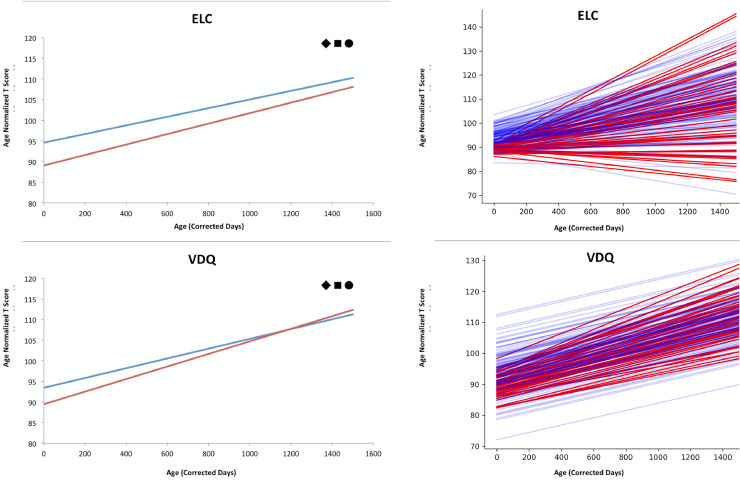
Longitudinal cognitive maturation trajectories of ELC, and VDQ. Carrier trajectories are denoted in red and non-carrier trajectories are displayed in blue. ◆ indicates a significant difference in baseline cognitive performance (*p* < 0.05, corrected for multiple comparisons), a • indicates that the Mullens composite score in our cohort showed a significant rate of change relative to the normal sample in the cognitive development model (*p* < 0.05, corrected for multiple comparisons), and a ■ indicates a significant difference in rate of cognitive development between APOE ε4 carriers and non-carriers (*p* < 0.05, corrected for multiple comparisons). NVDQ is not shown in this figure because this metric did not demonstrate a significant rate of change in our cohort relative to the normal sample. Adjacent to the population trajectories are individual trajectories of longitudinal cognitive development for ε4 carriers and non-carriers with carrier trajectories displayed in red and non-carrier trajectories displayed in blue.

Mixed effects linear models of Mullens composite scores was performed with ELC and VDQ showed a significant rate of development relative to the normal sample; while, NVDQ did not show significant trajectory of development. Group differences in baseline cognitive performance (intercept parameter) were observed between ε4 carriers and non-carriers, with ε4 carriers having significantly lower baseline NVDQ, ELC, and VDQ. Since NVDQ, for either ε4 carriers or non-carriers, does not demonstrate a significant rate of change in our cohort relative to the normal sample, the group difference in baseline cognitive performance extends across the entire age range of this cohort, with ε4 carriers showing significantly lower NVDQ. For ELC and VDQ, ε4 carriers displayed a significantly greater rate of cognitive development with trajectories revealing that VDQ in ε4 carriers eventually surpasses non-carriers. Cognitive maturation trajectories are displayed in Fig. 4. Post-hoc analysis of specific cognitive performance metrics revealed significant age associations with visual reception, expressive language, and receptive language development. For NVDQ, post-hoc analysis revealed significantly lower baseline fine motor and visual reception scores in ε4 carriers. Visual reception further showed a significantly greater rate of development in ε4 carriers despite no significant age association observed in NVDQ. For VDQ, post hoc analysis revealed significant differences in expressive language development, with ε4 carriers showing greater rate of development, but lower baseline scores. No significant differences in rate of receptive language development or baseline scores were observed. A complete list of model parameter comparisons is provided in Supplementary Table 4.

### 3.3 Analysis of the role of differential myelin development as a mediator for the effects of APOE genotype on cognitive ability

Using mixed effects modeling individual myelin parameters (*α, β, γ*, and *η*) were calculated for each subject in each of the neuroanatomical regions analyzed. Additionally, cognitive age association and intercept parameters for ELC, VDQ, and NVDQ were calculated for each subject. Mediation analysis was performed with myelin parameters as the mediator between APOE genotype and cognitive relationships. With regards to NVDQ, mediation analysis for the age association parameter was not performed due to prior results indicating that NVDQ does not have a significant relationship with participant age. Per the Baron and Kenny approach to identify a true mediation effect; APOE genotype status was shown to have a significant relationship to the examined cognitive metrics (see Fig. 4 and **Supplementary Table 2**); APOE genotype status was shown to be significantly associated with longitudinal myelin development parameters (see Figs. 2 and 3 and **Supplementary Table 1**); and in the combined model the APOE genotype to cognitive metric relationship was shifted (total effects < 0.05). With regards to ELC, mediation analysis for the ELC slope (age association) parameter did not show significance when both genotype and any of the four myelin parameters were combined into the same model (*p*-value for total effects was > 0.05), and was therefore not included. The remaining 4 analyses consisted of exploring the mediation effect of longitudinal myelin parameters (*α, β, γ*, and *η*) on APOE genotype to ELC intercept, NVDQ intercept, VDQ intercept, and VDQ slope relationships. Of note the longitudinal myelin parameters had both significant positive and negative mediation effects on the APOE genotype to cognitive score relationship.

**Fig. 5:**
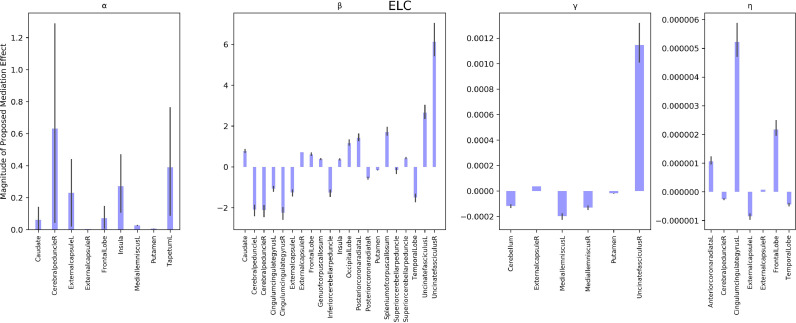
Mediation analysis of the role of myelin development parameters on ELC. Each panel refers to a different myelin development parameter. with the first panel revealing brain regions with the α myelin development parameter as a significant mediator; the second panel displays brain regions where the β myelin development parameter was a significant mediator, the third panel displays regions where the γ myelin development parameter was a significant mediator, and the fourth panel displays regions where the η parameter was a significant mediator. Each bar represents the magnitude of the proposed mediation effect for a specific neuroanatomical location and axis vary significantly between panels due to significant variation in parameter magnitude (see supplementary Table 2 for differences in myelin parameter magnitudes. Each bar represents the magnitude of the proposed myelin mediation effect for a specific neuroanatomical location. All neuroanatomical locations displayed significantly mediated the APOE genotype to ELC relationship.

**Fig. 6:**
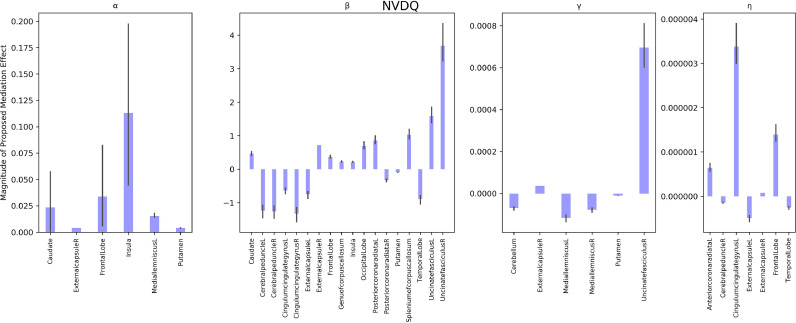
Mediation analysis of the role of myelin development parameters on NVDQ. Each panel refers to a different myelin development parameter, with the first panel revealing brain regions with the α myelin development parameter as a significant mediator; the second panel displays brain regions where the β myelin development parameter was a significant mediator, the third panel displays regions where the γ myelin development parameter was a significant mediator, and the fourth panel displays regions where the η parameter was a significant mediator. Each bar represents the magnitude of the proposed mediation effect for a specific neuroanatomical location and axis vary significantly between panels due to significant variation in parameter magnitude (see supplementary Table 2 for differences in myelin parameter magnitudes. All neuroanatomical locations displayed significantly mediated the APOE genotype to NVDQ relationship.

**Fig. 7:**
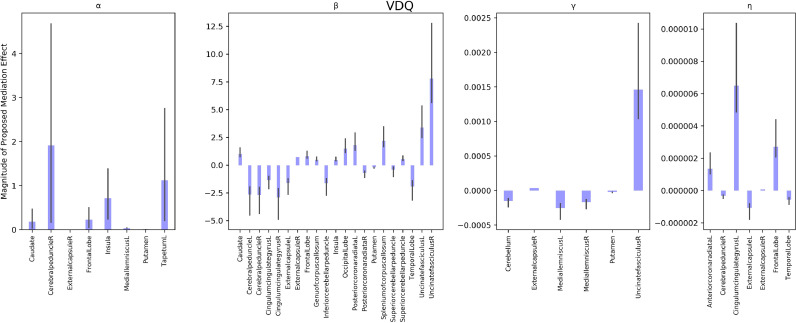
Mediation analysis of the role of myelin development parameters on VDQ. Each panel refers to a different myelin development parameter. with the first panel revealing brain regions with the α myelin development parameter as a significant mediator; the second panel displays brain regions where the β myelin development parameter was a significant mediator, the third panel displays regions where the γ myelin development parameter was a significant mediator, and the fourth panel displays regions where the η parameter was a significant mediator. Each bar represents the magnitude of the proposed mediation effect for a specific neuroanatomical location and axis vary significantly between panels due to significant variation in parameter magnitude (see supplementary Table 2 for differences in myelin parameter magnitudes. Each bar represents the magnitude of the proposed myelin mediation effect for a specific neuroanatomical location. All neuroanatomical locations displayed significantly mediated the APOE genotype to VDQ relationship.

APOE genotype to NVDQ intercept relationships were significantly mediated by the APOE genotype to α myelin development parameter relationship in multiple brain regions encompassing the insula, caudate, left medial lemniscus, and right external capsule; by the APOE genotype to β myelin development parameter in the bilateral cerebral peduncles, bilateral external capsules, frontal lobe, bilateral posterior corona radiate, and bilateral uncinate fasiculus; by the APOE genotype to γ myelin development parameter in the right external capsule and bilateral medial leminiscus; and by the APOE genotype to η myelin development parameter in the left cingulate gyrus, bilateral external capsule, frontal lobe, and right cerebral peduncle (see Fig. 5). APOE genotype to ELC intercept relationships were significantly mediated by differential myelin development with regards to the α parameter in the caudate, right cerebral peduncle, frontal lobe, insula; to the β parameter in the bilateral cerebral peduncles, bilateral external capsules, bilateral posterior corona radiata, and bilateral uncinate fasciculus; to the γ parameter in the bilateral medial lemniscus, putamen, and right uncinate fasciculus; and to the η parameter in the bilateral external capsule, right cerebral peduncle, and left cingulate gyrus (see Fig. 6). APOE genotype to VDQ intercept relationships were significantly mediated by differential myelin development with respect to the α parameter in the insula, left medial lemniscus, and right cerebral peduncle; with respect to the β parameter in the bilateral cingulate gyrus, bilateral cerebral peduncle, bilateral posterior corona radiata, and bilateral uncinate fasiculus; with respect to the γ parameter in the bilateral medial lemniscus, putamen, cerebellum, and right uncinate fasiculus; and with respect to the η parameter in the bilateral cingulate gyrus, bilateral external capsule, frontal lobe, and temporal lobe (see Fig. 7).

## 4 Discussion

In this study, we sought to characterize and compare the earliest longitudinal white matter differences between non-carriers and carriers of APOE ε4 allele, the major genetic risk factor for late-onset Alzheimer's disease.  Our findings demonstrate that ε4 carriers display significant MWF trajectory differences in all four parameters of early myelin development in multiple neuroanatomical locations (Sexton et al., 2011). Moreover, developmental trajectories of general cognition, verbal, and non-verbal ability were observed to differ between ε4 carriers and non-carriers, and these differences are significantly mediated by the differences observed in myelin development. These results are the first to show longitudinal brain and cognitive differences between ε4 carriers and non-carriers throughout infancy and early childhood, further demonstrating the importance of the APOE gene on early neurodevelopment.

Our group previously compared cross sectional MWF changes in 162 infants, 60 ε4 carriers and 102 non carriers, 2–25 months of age (Dean et al., 2014c) and found infant ε4 carriers to exhibit an attenuated relationship between MWF and age in extensive white matter tracts such as optic radiations, corticospinal tracts, and splenium of the corpus callosum, and a stronger relationship in more restricted, later developing frontal white matter regions in this cross-sectional analysis (Dean et al., 2014c). We also previously found that infant ε4 carriers, between 2 and 6 months of age, had reduced MWF than non-carriers in the precuneus, posterior/middle cingulate, lateral temporal, and medial occipitotemporal regions (Dean et al., 2014c).

Unlike our prior analysis in which brain differences between infant ε4 carriers and non-carriers were explored by comparing image voxels between the two groups, this study examined brain differences in pre defined neuroanatomical tracts, that our group has extensively characterized and mathematically modeled in healthy typically developing cohorts (Dean et al., 2014a). Furthermore, since our hypothesis was an exploratory analysis of differential longitudinal myelin and cognitive development in ε4 carriers, and that regions with cross sectional differences in brain anatomy, may or may not show differences in longitudinal brain development we chose to include all brain regions in this current study not eliminate any regions that previously did not show significance in our cross sectional analysis. Overall, our results did verify that regions such as the corticospinal tract, splenium of corpus callosum, and frontal white matter all of which as previously mentioned showed decreased myelin in ε4 carriers also demonstrated longitudinal differential myelin development, and further characterized specific differences in both rate onset of myelination and rate of myelination. Additionally our results revealed that regions such as the external capsule, tapetum, uncinate fasiculus, and medial leminiscus also show longitudinal differences in brain development that did not show cross sectional differences in infancy (Dean et al., 2014c). Interestingly, our longitudinal analysis revealed that over a larger age range (up to 2100 days of development), regions in which ε4 carriers previously had greater MWF (Dean et al., 2014c), have a decreased rate of MWF development that continues until 5.5 years of age. This decreased rate of development allows the non-carriers to “catch-up” and eventually surpass the ε4 carriers by approximately 3 years of age. This observation was fairly consistent across multiple brain regions and provides a unique perspective that while ε4 carriers might myelinate earlier, there slower early rate of development (decreased γ parameter) and there earlier transition in myelination (decreased α parameter) lead to this biological phenomena.

Variability of the white matter developmental trajectories of specific white matter regions and pathways, and the differences in rates of MWF development between ε4 carriers and non-carriers reveals the complex influence of the APOE gene on human brain development. As a protein that is essential in the transport and clearance of cholesterol, APOE has an important role in the development, maintenance, and repair of myelinated neurons (Hauser et al., 2011). Furthermore, having multiple isoforms, the APOE protein can be variable or deficit in functions involved in brain metabolism, inflammation, plasticity, beta amyloid accumulation and clearance, tau dephosporylation, and neurodevelopment (Mahley and Rall, 2000, Strittmatter and Roses, 1995). Our results raise new questions on how these biochemical and ultrastructural mechanisms may lead to ε4 carriers exhibiting differing rates of myelination in regions across the brain.

Another unique area of interest would be to examine myelin ultrastructure to observe differences between APOE genotypes. While MWF measurements from mcDESPOT are sensitive to myelin changes based on an increase in water trapped within the lipid layers as the myelin sheath accumulates (Deoni et al., 2008, Deoni et al., 2013), we are still limited at this time due to the resolution of our current imaging techniques in examining further ultrastructural geometric and biochemical differences such as myelin flattening and thickness of individual myelin layers compared to an increase in the number of lipid bilayers

Expanding upon our initial analysis, we also demonstrated that ε4 carriers show variable MWF development in brain regions that have previously been implicated in Alzheimer's Disease (Sexton et al., 2011). Onset of MWF development, rate of MWF development, and change from initial to later myelin development rate were found to differ between ε4 carriers and non-carriers in these regions including the right uncinate fasciculus and temporal lobe white matter, and the left occipital white matter, suggesting they experience complex differential neurodevelopment.

Our findings of altered white matter microstructure has been reported in cognitively unimpaired younger and older adult APOE ε4 carriers using diffusion tensor imaging (Chiang et al., 2012, Heise et al., 2011) and transverse relaxation rates (Bartzokis et al., 2006, Lu et al., 2011). For example, Heise and colleagues demonstrated that APOE ε4 carriers exhibit altered white matter structure as early as 20 years of age. Our findings suggest that such differences may already be present in early infancy. We further propose that differences between ε4 carriers and non-carriers exhibit a strong dependence on the trajectory of development, with white matter content and rate of white matter maturation differing between carriers and non-carriers. The current study raises new questions about how these variations in white matter development continue throughout life in ε4 carriers, and their implications in causality of AD pathology.

Prior longitudinal analysis on the influence of APOE ε4 genotypes on brain development in adolescents has been primarily restricted to measures of cortical thickness (Shaw et al., 2007). These results have shown decreases in cortical thickness within AD sensitive regions, such as the entorhinal cortex, in ε4 carriers (Shaw et al., 2007). Our results reveal that longitudinal differences in brain development extend beyond cortical thickness to the white matter microstructure. Future studies are needed to understand the interplay between cortical and myelin development, as these processes might be interdependent (Croteau-Chonka et al., 2016, Deoni et al., 2015).

Findings related to the association between APOE ε4 carrier status and cognition in infants, children, and adolescents is conflicting. Ihle and colleagues, in their meta-analysis, reported that small studies of ε4 positive healthy young adults and children, ages 5 to 35 years reveal slightly better cognitive performance with regards to executive tasks; while, other studies have shown slightly worse cognitive performance in ε4 carriers (Ihle et al., 2012). Our preliminary findings suggest slightly but significantly lower baseline cognitive scores in ELC, VDQ, and NVDQ composite measures of cognition in APOE ε4 carriers. No significant age associations were observed with NVDQ, the composite score of fine motor and visual reception skills and as a result ε4 carriers display slightly lower NVDQ scores over this age range. Early learning composite, a surrogate measure of childhood IQ, and VDQ, the composite score of expressive and receptive language both show an age association and significant greater rate in development. Unlike the work of others who have not considered longitudinal cognitive development (Wright et al., 2003), we find ε4 carriers to have a decreased longitudinal non-verbal cognitive performance, but an increased rate of overall cognitive development and verbal development. Post hoc analysis further classified the greater rate of verbal development to arise from a significantly greater rate of development in expressive language. The higher NVDQ cognitive scores observed in non-carriers were evident in both fine motor and visual reception, but unlike fine motor, visual reception did show a significant age association with ε4 carriers having a significantly greater rate of development. Additional studies are needed to confirm these findings in a larger number of APOE ε4 carriers, clarify the nature and persistence of cognitive changes, if any, and determine whether or not they have any clinical relevance. Prior studies have additionally observed a longitudinal age-genotype association in cognitive decline (Quiroz et al., 2013).

Unlike prior studies, this is the first paper to not only reveal a longitudinal myelin developmental differences in infancy and early childhood, but also that the longitudinal cognitive developmental differences we have observed can be explained by the myelin differences. Through the successful implementation of a true mediation analysis we show that the myelin development, specifically differences in the four myelin development parameters based on APOE genotype both positively and negatively mediates ELC, VDQ, and NVDQ scores. This provides significant insight that the presence of an altered longitudinal myelin trajectory in specific neuroanatomical locations provides insight for why ε4 carriers have an initial lower ELC, NVDQ, and VDQ scores. Interestingly, ε4 carriers demonstrate a greater rate of ELC change (greater slope), but this relationship was not significantly mediated by myelin development (addition of myelin caused the total effects of the model to have a *p* value > 0.05). Another unique phenomena observed during this analysis is the presence of negative mediation results. This result most likely indicates a significant suppression effect in which instead of the effects of APOE genotype on myelin development further strengthening the relationship of APOE genotype to cognitive development, the effects of APOE genotype on myelin development actually reduce the relationship of APOE genotype on cognitive development. Reasons for this are still unclear and we are unaware of any underlying biologic process or evolutionary mechanism that contributes to or explains this finding. As the first study to observe this relationship we hope that further work will continue to explore the role of myelin development as a mediator between APOE genotype and cognitive performance.

This study has several limitations. The analysis of differential brain development was restricted to only myelin development and future studies are needed to expand these results to encompass differences in gray matter development between APOE genotypes as well as understand correlations between myelin and gray matter development. Additionally, differences in both myelin development and cognitive development were statistically significant, but these changes have not yet been shown to be clinically significant or even permanent. Another potential limitation to this study is that differences in myelin and cognitive development were only analyzed between APOE ε4 carriers and non-carriers. APOE genotype also includes additional polymorphisms such as ε4/ε4, ε2/3, ε2/ε4. While these genotype combinations are rare, we do believe that future analysis further subdividing participants in 4+ genotype groupings will hopefully provide greater insight on the effects of APOE on early neurodevelopment.

Evaluation of neurodevelopment and MWF through MR imaging has inherent limitations. While some literature has questioned that mcDESPOT might provide inconsistent MWF estimates (West et al., 2019), multiple prior studies have demonstrated mcDESPOT to be not only an effective and sensitive technique for MWF ascertainment (Deoni et al., 2008, 2013, O'Muircheartaigh et al., 2019, Deoni and Kolind, 2015, Kolind et al., 2012), but also a robust indicator of the dynamic change during early brain development (Deoni et al., 2012, Dean et al., 2014a, 2014d). Specifically a recent study demonstrated that MWF derived from mcDESPOT when compared to MWF from T2-relaxation with combined Gradient and Spin Echoes, magnetization transfer imaging magnetization transfer ratios, and T1 relaxation quantitive T1; MWF from mcDESPOT demonstrated the best accuracy in terms of statistical sensitivity to WM lesions in MS (O'Muircheartaigh et al., 2019). Therefore, we believe that the use of mcDESPOT for evaluation of early myelination is a sensitive and accurate technique.

As a study that considers the role of APOE genotype of myelin and cognitive development there is always a risk of additional unaccounted confounders that could bias the relationships that we report. Through extensive identification of demographic information along with participant medical and perinatal history (see methods and Table 1) we have attempted to control for such confounders. Nevertheless, future studies are needed to further explore the role of additional co-variates and how they might influence the relationship between APOE and myelin or cognitive development.

Moreover, a limitation in this study includes the exclusion of participants with age greater than 1500 days from the cognitive analysis. This decision was necessary as cognitive changes appeared to be static after 1500 days and therefore did not fit in the linear mixed effects modeling frame work. While there is value in observing cognitive differences in this time period it does alter our models if it is included and therefore requires a separate analysis for comparison. As stated previously in the mediation analysis, all imaging data points were also restricted to 1500 days of age and all cognitive assessments occurred within 1 week of obtaining MRI data.

This study is the first longitudinal study of differential white matter development based on APOE genotype, and as such should be viewed as preliminary, emphasizing the need for longer studies to understand the impact and permanence of the observed brain differences.

## 5 Conclusions

Overall, this longitudinal study supports the possibility that APOE ε4 carriers have altered trajectories of white matter and cognitive development in early childhood, and it continues to raise questions about the role of APOE in normal human brain development, the relevance of these developmental changes to the predisposition to AD, and how such brain changes early in life may lead to subsequent AD pathology.

## CRediT authorship contribution statement

**Justin Remer:** Conceptualization, Software, Formal analysis, Investigation, Data curation, Writing - original draft, Writing - review & editing, Methodology. **Douglas C. Dean:** Conceptualization, Software, Formal analysis, Investigation, Data curation, Writing - original draft, Writing - review & editing, Methodology. **Kewei Chen:** Conceptualization, Writing - review & editing, Supervision, Project administration. **Rebecca A. Reiman:** Data curation, Resources. **Matthew J. Huentelman:** Data curation, Conceptualization, Project administration, Writing - review & editing. **Eric M. Reiman:** Conceptualization, Data curation, Writing - review & editing, Supervision, Funding acquisition, Project administration. **Sean C.L. Deoni:** Conceptualization, Methodology, Software, Formal analysis, Investigation, Data curation, Writing - review & editing, Supervision, Funding acquisition, Project administration.

## Appendix Supplementary materials

Image, application 1

Image, application 2

Image, application 3

Image, application 4
