# Supplementary material for: Longitudinal white matter and cognitive development in pediatric carriers of the apolipoprotein ε4 allele
Source: Neuroimage. 2020 Nov 15;222:117243. doi: 10.1016/j.neuroimage.2020.117243 (PMC7779366; doi:10.1016/j.neuroimage.2020.117243)
Supplement: Supplementary file 1 [file mmc1.docx]

**Online Supplementary Appendix**

**Methods:**

**Pediatric MRI Data Acquisition**

MRI data was acquired during natural, non-sedated sleep for children less than 4 years of age; while older children were scanned while watching a favorite movie or TV show. Scanning noise levels were minimized to ensure sleeping children remained asleep and awake children were comfortable throughout the entire scan. Noise reductions were achieved by reducing the imaging slew rates and gradient amplitudes to 15mT/m/s and 30mT/m, respectively. Further noise reduction measures included the use of electrodynamic headphones (MR Confon, Germany) and a removable sound insulating foam insert (Ultra Barrier HD Composite, UltraBarrier USA) that was fitted inside the bore. Swaddling children with MedVac immobilization bags and memory foam cushions positioned around the child’s head minimized subtle head/body movements.

Optimized, whole-brain (1.8x1.8x1.8 mm^3^) mcDESPOT protocols consisted of 8 T_1_-weighted spoiled gradient echo (SPGR or spoiled FLASH) images, 2 inversion-prepared (IR)-SPGR images, and 16 T1/T2-weighted balanced steady-state free precision (bSSFP or TrueFISP) images. SPGR and bSSFP images were acquired at varying flip angles and the bSSFP were collected at two phase increments of 0 and 180 degrees^9^. Myelin water fraction (VF_M_) values, a surrogate metric of myelin content, were calculated at each image voxel by fitting the SPGR and bSSFP data to a 3-pool tissue model^9,13,31^

**mcDESPOT Processing**

Following image acquisition, individual SPGR, IR-SPGR, and bSSFP images for each participant were linearly co-registered to take into account possible subtle head movements, and non-parenchyma voxels were removed through a deformable model approach. A novel imaging technique, termed Multicomponent Driven Equilibrium Single Pulse Observation of T_1_ and T_2_ (mcDESPOT), was used to derive myelin water fraction (MWF) measurements, a surrogate metric of myelin content, at each imaging voxel by fitting a three-pool multicomponent relaxometry model to the SPGR and bSSFP image data^9,13^. This three-pool model estimates the volume fractions and relaxation times for the extra/intracellular water, non-exchanging free water, myelin associated water and provides specific characteristics regarding the microstructural water pools within brain tissue^13,31^. As a result, these measures provide more detailed and specific information regarding the neurological microstructure.

**Longitudinal Data Registration**

To reduce inconsistencies associated with independently registered longitudinal data a longitudinal registration pipeline was used that first align generates a T1 weighted template from the acquired longitudinal time points using symmetric diffeomorphic normalization and a cross-correlation similarity metric^10,15^. An initial rigid registration is performed between the subject’s high flip angle SPGR images and a study specific T1-weighted template, generated from children of the same age range to bring the images into a rough alignment^9,10^. The subject specific template was then nonlinearly registered to the study template using symmetric diffeomorphic normalization, and then individual parameter maps for each time point were transformed to the common study template by concatenating the previously stated transformations in a single interpolation step^10^. Registration was performed with the Advanced Normalization Tools (ANTs) software package and a T_1_-weighted study specific pediatric brain template^9,10,15^. Registration was initially performed using a T_1_-weighted SPGR image, with the calculated image transformations subsequently applied to the quantitative MWF map. After successful registration, a conservative 3-mm Gaussian kernel was applied to smooth the MWF maps in order to accommodate residual subtle structural variations among the individual subjects.

**Region of Interest Analysis**

To analyze myelination in specific brain regions and specific white matter tracts, we employed a region of interest (ROI) analysis using the FMRIB Software Library (FSL). Anatomically co-registered masks obtained from the MNI database and the John Hopkins diffusion tensor imaging (DTI) based white matter atlas were overlaid onto longitudinally registered mcDESPOT MWF maps^32,33^. Mean values from each ROI were computed, allowing myelin content of the brain regions and white matter tracts to be quantified for every subject and for each genotype. Due to known brain asymmetry and asymmetry involved in myelination, left and right brain regions were considered independently^9,10,34^. In total, 66 regions were examined and developmental trajectories for each region were constructed by plotting mean VF_M_ against age.

**Modeling Brain Development**

In order to be consistent with our previous cross-sectional analysis of MWF development during the first 2 years of life, MWF we utilized a logarithmic growth model:

MWF(age) = α_l_ ln(x) + β_l_  Eq. 1

Where α_l_ is a parameter that reflects the rate of MWF development (i.e. slope) and β_l_ controls for initial MWF (i.e. intercept).

However, the development of MWF across early childhood is highly nonlinear and has been shown to be best characterized by a modified Gompertz growth model^10^. Therefore, in order to expand the analysis to a larger age range (across the first 2100 days of development), we utilized this previously described model:

MWF(age) = α*exp(-exp(β-γ*age) + η*age) Eq. 2

The four parameters associated with this model offer intuitive detail on the biological mechanisms of MWF development^10,16^. For example, α influences for the overall size of the curve, β controls for the initial lag of the growth curve, and γ and δ relate to the rate of development. In the nonlinear mixed-model representation, these four parameters (α,β,γ,δ) encompass the sum of the fixed and random effects, where fixed effects describe the population MWF development, and the random effects relate to subtle individual variation of MWF trajectories . Non-linear mixed effect regressions were generated separately for subjects who contained at least one copy of the ε4 allele (carriers) and for subjects who had no copies of the ε4 allele (the non carriers).

Cognitive development trajectories for ε4 carriers and non carriers were generated using linear mixed effects modelings:

Mullens Composite Cognitive Score = α*age + β + b Eq. 3

Where each group was modeled with its own developmental trajectory.
